# Characteristic Analysis and Health Risk Assessment of PM_2.5_ and VOCs in Tianjin Based on High-Resolution Online Data

**DOI:** 10.3390/toxics12090622

**Published:** 2024-08-23

**Authors:** Yanqi Huangfu, Feng Wang, Qili Dai, Danni Liang, Guoliang Shi, Yinchang Feng

**Affiliations:** 1 State Environmental Protection Key Laboratory of Urban Ambient Air Particulate Matter Pollution Prevention and Control, College of Environmental Science and Engineering, Nankai University, Tianjin 300350, China; hfyq1993@hotmail.com (Y.H.); wfeng@email.tjut.edu.cn (F.W.); daiql@nankai.edu.cn (Q.D.); nksgl@nankai.edu.cn (G.S.); 2 CMA-NKU Cooperative Laboratory for Atmospheric Environment-Health Research (CLAER/CMA-NKU), Tianjin 300350, China; 3 School of Environmental Science and Safety Engineering, Tianjin University of Technology, Tianjin 300384, China

**Keywords:** PM_2.5_, VOCs, online data, source apportionment, health risk assessment

## Abstract

This study leveraged 2019 online data of particulate matter (PM_2.5_) and volatile organic compounds (VOCs) in Tianjin to analyze atmospheric pollution characteristics. PM_2.5_ was found to be primarily composed of water-soluble ions, with nitrates as the dominant component, while VOCs were predominantly alkanes, followed by alkenes and aromatic hydrocarbons, with notable concentrations of propane, ethane, ethylene, toluene, and benzene. The receptor model identified six major sources of PM_2.5_ and seven major sources of VOCs. The secondary source is the main contribution source, while motor vehicles and coal burning are important primary contribution sources in PM_2.5_. And, industrial processes and natural gas volatilization were considered major contributors for VOCs. A health risk assessment indicated negligible non-carcinogenic risks but potential carcinogenic risks from trace metals As and Cr, and benzene within VOCs, underscoring the necessity for focused public health measures. A risk attribution analysis attributed As and Cr in PM to coal combustion and vehicular emissions. Benzene in VOCs primarily originates from fuel evaporation, and industrial and vehicular emissions. These findings underscore the potential for reducing health risks from PM and VOCs through enhanced regulation of emissions in coal, industry, and transportation. Such strategies are vital for advancing air quality management and safeguarding public health.

## 1. Introduction

With the swift progression of urbanization, an array of pollutants, including particulate matter and VOCs, have been introduced into the atmosphere, exacerbating air pollution issues in numerous urban centers across China [1]. In response, the initiation of the Air Pollution Prevention and Control Action Plan (APPCAP) in 2013 marked a significant regulatory effort to mitigate these emissions [2,3]. Consequently, there has been a notable reduction in anthropogenic atmospheric particulates and inhalable particulate matter, as indicated by substantial decreases in particulate matter concentrations [2,4,5]. Despite the overall particulate pollution having been significantly improved, the problem of secondary pollution has gradually become prominent. Additionally, the emergence of ozone pollution has introduced a new environmental challenge. VOCs have been identified as key precursors in the formation of O_3_ and secondary organic aerosols (SOAs), which are integral to fine particulate matter and significant contributors to haze events [6,7]. The public health implications of particulates and VOCs have risen to prominence as a pivotal area of research [8,9,10].

VOCs originate from diverse sources, spanning both anthropogenic activities and natural processes [11,12]. The intensification of human activities has led to a continuous increase in the anthropogenic emissions of VOCs. Research on the impact and health risks of VOCs emitted from specific industries is a burgeoning field, with the latest references indicating a growing focus on this area [13,14,15]. In recent years, there has been a surge in studies aimed at tracing the sources of VOCs in the atmospheric environment. The Positive Matrix Factorization (PMF) model has been extensively applied in studies aimed at identifying sources of VOCs [16]. Chronic exposure to certain toxic VOCs at high concentrations poses significant health risks, including sensory irritation, fatigue, neurological damage, and carcinogenesis [17,18,19,20,21]. Researchers have utilized methodologies aligned with USEPA standards to assess VOC-related health risks in certain regions of India [22,23], Canada [24], and China [25,26].

The concentration of atmospheric particulate matter has seen a marked decline, with current pollution primarily attributed to secondary pollutants. During periods of severe pollution, secondary pollutants can account for approximately half of the total PM_2.5_ load. The health risks associated with particulate matter are largely due to trace metal elements such as Pb, Mn, Ni, Cr, Cd, and Hg. These elements possess pathophysiological toxicity and are linked to a range of severe health hazards, including respiratory inflammation, cardiovascular and pulmonary diseases, and DNA damage [27]. They have also been shown to induce severe cardiovascular and central nervous system disorders [28,29]. Cd toxicity is associated with lung cancer, hepatic and renal pathologies, osteocalcin, and dermatological conditions [30]. Ni and V, emitted through fuel combustion, have a positive correlation with human cardiovascular and respiratory diseases [31,32,33]. Excessive Ni intake can lead to respiratory ailments [34], while Pb overexposure can impair the nervous system, endocrine and immune functions, skeletal development, and enzymatic cycles [35]. Mn has been identified as a potential neurotoxin and nephrotoxin.

This research delves into an integrated analysis of PM and VOCs in Tianjin for the year 2019, leveraging the online monitoring data from the advanced Super Observation Station located in Jinnan District. It presents a detailed characterization of the concentration and compositional profiles of PM and VOCs, offering insights into the chemical signatures of air pollutants within the region. The principal contributors of PM and VOC pollution have been identified in this area based on the widely used PMF model. Furthermore, this study conducts a thorough assessment of the health impacts associated with the identified PM and VOC components. By quantifying the exposure levels and evaluating the associated health risks, it pinpoints the predominant sources contributing to health risks, providing critical insights into the environmental and public health challenges posed by these pollutants.

## 2. Materials and Methods

### 2.1. Sampling and Chemical Analysis

Data collection was conducted at the Nankai University Air Quality Research Supersite (NKAQRS), situated at 38.99° N latitude and 117.33° E longitude, within the Jinan District of the Tianjin campus. The monitoring period spanned from 1 January to 31 December 2019. The NKAQRS is adjacent to a student dormitory area, with a low-traffic road located 20 m to the north. The winds predominantly blew from the east and southeast throughout this study. This study recorded a mean wind speed (WS) of 1.5 m/s; an average temperature of 14.5 °C; and a mean relative humidity (RH) that fluctuated between 14.1% and 98.7%, averaging 58.4% for the year 2019.

Despite the synchronization of the majority of data across the years, occasional gaps were observed due to instrument malfunctions or unforeseen events. The PM_2.5_ mass concentration was continuously tracked using a BPM-200 Beta Particulate Monitor (Focused Photonics Inc., Hangzhou, China). Meteorological parameters, such as Temp, RH, and solar radiation, were captured by a compact weather station (WS600-UMB, LUFFT Inc., Fellbach, Germany).

All particulate matter instruments were integrated with a PM_2.5_ inlet to ensure accurate and targeted sampling of fine particulate matter. Concentrations of elements, including K, Ca, Ti, Cr, Mn, Fe, Ni, Cu, Zn, As, Ba, and Pb were meticulously monitored on an hourly basis utilizing an in situ X-ray fluorescence analyzer (AMMS-100, Focused Photonics Inc., Hangzhou, China). Concurrently, water-soluble ions such as SO_4_^2−^, NO_3_^−^, NH_4_^+^, Cl^−^, Na^+^, K^+^, Mg^2+^, and Ca^2+^ were precisely quantified through an in situ ion chromatography system (URG 9000D, Thermo Fisher Scientific Inc., Waltham, MA, USA). Additionally, Organic Carbon (OC) and Elemental Carbon (EC) were continuously assessed hourly with a sophisticated thermal-optical carbon analyzer (OCEC-100, Focused Photonics Inc., Hangzhou, China). Elements K and Ca were selected to be included when constructing the PM_2.5_ dataset.

In total, 54 VOC species across four sub-categories—27 alkanes, 10 alkenes, 16 aromatic hydrocarbons, and 1 alkyne (acetylene)—were recognized as photochemical precursors by the United States Environmental Protection Agency (US EPA). The analyses were executed using a photoionization detector (PID) and a flame ionization detector (FID), ensuring high sensitivity and effective identification (GC955-611/811, Synspec B.V., Groningen, Netherlands). It should be noted that due to the large range of missing values of styrene, although it has a more important indicative role, it was still removed from the overall system.

Quality assurance and quality control (QA/QC) measures were implemented based on previous studies [36,37,38] to ensure the reliability of the collected data. For PM_2.5_ monitoring, it was meticulously maintained through monthly cleaning and calibration of the cutting head, as well as calibration of the sampling flow rate. The allowable deviation between the instrument’s indicated flow rate and that of the flowmeter was within ±2%. Single-point calibration was performed monthly, with multi-point calibration conducted semi-annually for the VOC analysis. The calibration curve had to demonstrate a correlation coefficient greater than 0.95 for at least 95% of the species. Ion calibration was adjusted every 1~2 months based on changes in the peak retention time, employing a multi-point calibration across six concentration gradients. The resulting standard curves for anions exhibited a correlation coefficient above 0.99, while for cations, all except sodium ions demonstrated a correlation coefficient above 0.95. The elemental analysis was subjected to annual verification against standard membrane filters, with the verification outcomes ensuring that the concentration discrepancies for all elements were within ±2% of the standard membrane filter concentrations. OC/EC calibration was performed annually or following the replacement of the helium–methane calibration gas. A multi-point calibration was executed with five points, where the correlation coefficient exceeded 0.95.

### 2.2. PMF Model

The EPA PMF 5.0 model served as the analytical framework for the source apportionment of PM2.5 and VOCs. Mathematically, the PMF model is represented as follows [39,40]:

(1) $$X_{ij}=\sum_{k=1}^{p}g_{ik}\cdot f_{kj}+e_{ij}$$

In this equation, $X_{ij}$ denotes the concentration of the *j*th species in the ith sample, $g_{ik}$ signifies the contribution of the *k*th source to the *i*th sample, $f_{kj}$ is the profile of the *j*th species in the kth source, $e_{ij}$ is the residual error for the *j*th species in the *i*th sample, and *p* refers to the total number of sources identified. The source contribution matrix and source profile matrix are the set of all $g_{ik}$ and $f_{kj}$ together.

The objective of employing the PMF model is to minimize the objective function $Q\left(E\right)$, defined as follows:

(2) $$Q\left(E\right)=\sum_{i=1}^{n}\sum_{j=1}^{m}{\left(\frac{e_{ij}}{\sigma_{ij}}\right)}^{2}$$

$\sigma_{ij}$ means the uncertainty for the *j*th species in the *i*th sample. Here, uncertainty is an important part of the input of the PMF model. Zeroes and negatives are not permitted for either the detection limit or the percent uncertainty. If the concentration is less than or equal to the Method Detection Limit (*MDL*) provided, the uncertainty is calculated using a fixed fraction of the *MDL* (Equation (3)). If the concentration is greater than the *MDL* provided, the calculation is based on a user-provided fraction of the concentration and *MDL* (Equation (4)).

(3) $$Unc=\frac{5}{6}\times MDL$$

(4) $$Unc=\sqrt{{\left(Error\;fraction\times Conc.\right)}^{2}+{\left(0.5\times MDL\right)}^{2}}$$

where the Error fraction (EF) encapsulates the aggregate deviations that occur throughout the entire experimental procedure, commencing with instrumental sampling and culminating with completion of the sample analysis. This encompasses potential sources of error such as instrumental instability, operational variability, and other elements that could compromise data precision. While specific EF values are often not explicitly stated in the majority of studies, a general range of 0.1 to 0.5 is commonly adopted as a reference [41].

Factor identification stands as a critical phase in the PMF analysis process, entailing the correlation of model-resolved factors with tangible sources of pollution. Employing marker species for this purpose is a method broadly recognized and utilized across the field. Our research approaches factor identification by drawing upon and consolidating insights gleaned from existing scholarly works.

### 2.3. Health Risk Evaluation of VOCs

The potential inhalation risks were quantified by integrating toxicity values with exposure estimates. The US EPA Inhalation Unit Risk (IUR) and Reference Concentrations (RfCs) were utilized to assess the carcinogenic and non-carcinogenic risks associated with each VOC species, providing insights into the actual exposure levels for workers in operational environments [42].

(5) $$EC=\frac{CA\times ET\times EF\times ED}{AT}$$

where EC represents the exposure concentration, CA is the ambient VOC concentration, ET means the daily exposure duration, EF refers to the exposure frequency per year, ED denotes the exposure duration in years, and AT is the average time for exposure calculations. The values of ET, EF, ED, and AT are 8 h/day, 365 day/year, 70 yr, and (70 × 365 × 24) h, respectively.

The non-carcinogenic risk was evaluated using the hazard quotient (*HQ*):

(6) $$HQ=\frac{EC}{RfC\times 1000}$$

The RfC values for specific VOCs were sourced from databases such as the Integrated Risk Information System (IRIS), the United States Agency for Toxic Substances and Disease Registry, or the California Office of Environmental Health Hazard Assessment (OEHHA) (Table S1).

The Hazard Index (HI), which is the sum of HQs for multiple pollutants, was used to assess the overall non-carcinogenic risk:

(7) $$HI=\sum HQ_{i}$$

For carcinogenic risks, the risk was calculated as follows:

(8) Risk=EC×IUR

Here, IUR represents the unit risk for a specific VOC species, which was also sourced from IRIS and OEHHA.

### 2.4. Health Risk Evaluation of Trace Metals

Drawing upon the research findings from the USEPA IRIS and the International Agency for Research on Cancer (IARC), health risks were categorized into carcinogenic and non-carcinogenic effects [43]. The health risk assessment model recommended by USEPA was employed, with the following computational approach:

(9) $$ADD(LADD)=\frac{C_{i}\times IR\times EF\times ED}{BW\times AT}$$

where ADD is the average daily dose for non-carcinogenic metals; LADD is the lifetime average daily dose for carcinogenic metals; $C_{i}$ is the concentration of trace elements in PM fractions; IR refers to the ingestion rate; EF represents exposure frequency; ED means the exposure duration; BW denotes the average body weight; and AT is the average time for exposure. The values of IR, EF, ED, BW, and AT are 20 m^3^/day, 350 day/year, 24 yr, 70 kg, and 24 × 365 days for non-carcinogens and 70 × 365 days for carcinogens.

Health risks were quantified using the hazard quotient (HQ) for non-carcinogenic effects and the incremental lifetime cancer risk (ILCR) for carcinogenic effects:

(10) $$HQ=\frac{ADD}{RfD}$$

(11) ILCR=LADD×CSF

Here, RfD denotes the reference dose, and CSF means the cancer slope factor. The parameters for these equations were obtained from the USEPA (Table S2). An HQ value greater than one for individual trace elements or the cumulative HQ indicates a potential for non-carcinogenic effects due to exposure. The acceptable risk level for carcinogenic effects was set at 10^−6^, representing a one in one million lifetime cancer risk.

### 2.5. Health Risk of Sources

Firstly, we needed to obtain the concentration contributions of every factor in the PMF. Subsequently, these contributions were multiplied by every species with a health risk present within the profiles of each factor. These results elucidated the specific contributions of various factors to the concentration of health risk species.

Second, with the obtained factor-specific contribution concentrations of health risk species, the health risk assessment formulas delineated in Section 2.3 and Section 2.4 were integrated. This integration facilitated the quantification of health risks attributable to the presence of these species within each factor. The aggregate of these health risks across all factors for a particular species was the total health risk of the species. Furthermore, the ratio of the health risk attributable to each factor relative to the total health risk needed to be calculated.

Finally, we obtained the source apportionment of health risk for each health risk species building upon the outcomes of the preceding step. These results could be used to evaluate the health risk of the sources.

## 3. Results and Discussion

### 3.1. Characteristics of PM_2.5_ and VOCs in Tianjin

**Figure 1:**
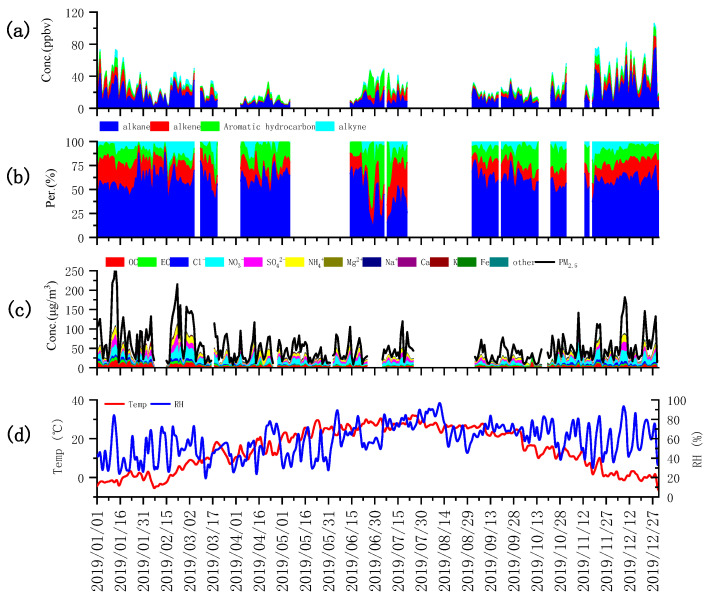
Time series of concentrations (**a**), percentages (**b**) of VOCs, aerosol concentrations and compounds (**c**) and meteorological parameters (**d**).

The year 2019 witnessed the delineation of total volatile organic compound (TVOC) concentrations and their respective proportions, as meticulously detailed in Table S3 and illustrated in Figure 1a,b. The average TVOC concentration peaked at 24.2 parts per billion by volume (ppbv), accompanied by a standard deviation of 22.5 ppbv. The mean concentrations for alkanes, alkenes, aromatics, and alkynes were recorded as 15.0, 4.8, 2.3, and 2.2 ppbv, respectively. These figures correspond to proportions of 61.9%, 19.8%, 9.4%, and 8.9%. of the total TVOC, respectively. Alkanes notably dominated with a significant edge in both concentration and proportion over other VOC subclasses. Propane and ethane emerged as the predominant alkane species, with concentrations of 4.6 ppbv (30.4%) and 2.9 ppbv (19.1%), respectively. Their origins can be primarily traced back to the evaporation and incomplete combustion of liquefied petroleum gas (LPG) and natural gas (NG), underscoring the substantial influence of NG or LPG in the area. The diagnostic ratio of isobutane to n-butane, standing at 0.7, highlights the notable impact of natural gas emissions, while the iso-pentane to n-pentane ratio of approximately 1.7 signaling the significant influence of gasoline evaporation. Among the alkenes, ethylene was found to have the highest proportion, accounting for 54.8%. In the aromatic category, toluene and the combined proportion of m-/p-xylene were the most prevalent, representing 28.9%, 29.0%, and 20.0%, respectively. The collective concentration of benzene, toluene, ethylbenzene, and xylene (BTEX) reached 2.1 ppbv, which encompasses 95.3% of the total aromatic content. This prevalence likely mirrors the substantial influence of solvent usage and vehicular emissions within the region. The toluene-to-benzene (T/B) ratio, with a value of 1.0, indicates that emissions from combustion processes are a source that merits considerable attention. In this study, the alkanes claimed the largest share of the VOC composition, succeeded by alkenes and aromatics. These findings are congruent with prior research conducted by Liu et al. [44]. and Li et al. [45] in the Tianjin and Beijing–Tianjin–Hebei (BTH) regions. However, it is important to acknowledge the marked variability in the primary VOC species across different regions within China. In some locales, aromatics hold a significant, if not dominant, position, as reported by Zhang et al. [46]. This underscores the geographically dependent nature of air pollutant composition and the necessity for localized air quality management strategies.

This 2019 study presents a detailed analysis of particulate matter concentrations and their chemical composition within the research area, as elegantly illustrated in Figure 1c. The effective online monitoring revealed a particulate matter concentration of 54.7 ± 50.7 μg/m^3^. Within this matrix, water-soluble ions predominated, constituting 39% of the particulate load, trailed by carbon components at 12%, elemental constituents at 4%, and a diverse array of other components that account for 45%. NO_3_⁻ was identified as the principal component of the particulate matter, with a concentration of 8.7 μg/m^3^. The remaining constituents are sequentially ordered by their abundance, including NH_4_⁺, SO_4_^2^⁻, OC, EC, Cl⁻, Na⁺, K, Fe, Ca, Zn, Mn, and others. The compositional analysis underscores the predominance of secondary particulate matter-related components, highlighting the imperative to address secondary pollution, particularly the formation of secondary nitrates. OC and EC are correlated with combustion sources, indicating that emissions from motor vehicles and coal combustion continue to be pivotal contributors to primary emissions. Tianjin, situated as a coastal city, exhibits elevated levels of Cl⁻, likely attributed to the influence of marine aerosols. Moreover, chloride emissions have also been associated with combustion processes. The presence of elements such as Fe, Ca, and Mn may be indicative of crustal dust contributions, while these elements, along with others, also suggest a linkage to industrial emissions.

### 3.2. Source Apportionment of PM_2.5_ and VOCs

**Figure 2:**
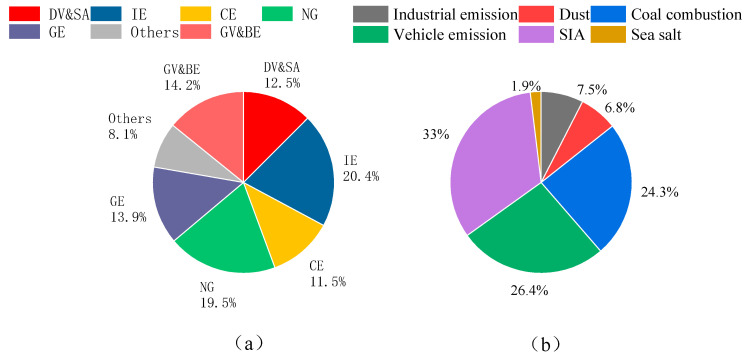
Source contribution of VOCs (**a**) and PM_2.5_ (**b**) in Tianjin.

The PMF model was used to analyze the sources of VOCs and particulate matter. The species included in the model needed to be screened, guided by stringent criteria: (1) the presence of species unique to each source profile, (2) their prevalence at elevated concentrations, (3) the capacity to compensate for potential sample volatilization, (4) the exclusion of highly reactive species that could skew results, and (5) an adequate representation of species to authentically reflect the actual source apportionment. The results are shown in Figure 2.

For VOCs, we meticulously selected 31 VOCs for the source contribution analysis. Following a meticulous evaluation process, we identified seven distinct factors for the PMF calculations, with their profiles for the year 2019, elegantly depicted in Figure 2a and Figure S1. Factor 1 is distinguished by its significant loading of aromatic hydrocarbons, including o-xylene, m-/p-xylene, toluene, ethylbenzene, and 1,2,4-trimethylbenzene. Notably, substantial concentrations of n-nonane, n-heptane, and n-hexane were also detected. C_6_ aromatics such as 1,2,4-trimethylbenzene and 1,3,5-trimethylbenzene are predominantly traced back to organic solvents utilized in the coating industry [47]. Trimethylbenzenes are established as principal constituents in a vast array of solvents employed across manufacturing sectors [48]. Toluene and ethylbenzene are byproducts of organic solvent applications [49]. The presence of long-chain alkanes, namely n-nonane, n-heptane, and n-hexane, in diesel exhaust fumes positions them as quintessential markers for diesel vehicle emissions [50]. Consequently, Factor 1 is delineated as an amalgam of diesel vehicle and solvent application (DV&SA). In Factor 2, we observed pronounced levels of ethylene and propylene, alongside n-pentene. Ethylene and propylene are emblematic of the petrochemical industry [51]. n-pentane as a key ingredient in the production of polystyrene foam, further substantiates the industrial linkage [49]. Thus, Factor 2 is demarcated as an industrial emission (IE). Factor 3 is emblematic of acetylene, recognized for its signature association with combustion sources [52]. Acetylene’s presence is intrinsically linked to the phenomenon of incomplete combustion predominantly emanating from the exhaust of internal combustion engines [53]. Accordingly, Factor 3 is categorized under combustion emission (CE). Factor 4 is characterized by ethane, a species quintessential to natural gas, thereby being classified as an NG volatilization (NG). Factor 5 exhibits a high concentration ratio of isobutane, 2-methylpentane, and 3-methylpentane, along with 1-butene. 2-methylpentane as a species is predominantly associated with gasoline-fueled vehicles, 1-butene primarily to gasoline volatilization [54], and C_4_-C_7_ alkanes as the principal volatiles in the gasoline headspace [50], thereby cementing the identification of Factor 5 as gasoline evaporation (GE). Factor 6 is marked by elevated concentrations of methylcyclohexane, accompanied by a notable presence of alkanes, alkenes, and aromatic hydrocarbons. While methylcyclohexane is recognized as a significant species for diesel volatilization, the industrial fingerprint of the accompanying species remains ambiguous. Thus, Factor 6 is posited as an ‘others’ category that includes diesel evaporation. Factor 7 is predominantly associated with isoprene, which constitutes 60% of its profile and stands as a significant bio-emission indicator [55]. Consequently, it is classified as a biogenic emission source. The high percentage of C_4_-C_6_ low-carbon alkane species, including pentane, butane, 3-methylhexane, 1,3-butadiene, 2,3-dimethylbutane, and cyclohexane, further corroborates the vehicular exhaust signature [56]. Therefore, Factor 7 is identified as a composite source of gasoline vehicle emissions and biogenic emission (GV&BE). In general, IE was responsible for the largest fraction of TVOC mass on average (20%), followed by NG (19%), GV&BE (14%), GE (14%), DV&SA (13%), CE (12%), and others (8%).

Twenty strictly screened components were incorporated into the PMF model for particle source analysis, and six factors were analyzed (Figure S2). The source contributions are elegantly portrayed in Figure 2b. The first factor, composed of Mn, Fe, Zn, Pb, OC, and Cl^−^, is attributed to industrial sources. Fe and Mn originate from emissions during the steel smelting process [57], while Zn and Pb are associated with non-ferrous metal processing, such as emissions from galvanization [2]. Cl^−^ is likely emitted from the production and use of chlorine-containing products or combustion emission. After all, Tianjin has long been renowned for its developed industry. The second factor consists of Ca, Ti, Fe, Mn, and Ba. These components are widely present in crustal dust; thus, this factor is attributed to urban dust resuspension sources. The third factor includes OC, EC, NH_4_^+^, SO_4_^2−^, As, Fe, Cu, and Zn and is assigned to coal combustion. Coal is the primary energy source for space heating in northern China during the cold season, and its combustion typically results in emissions of OC and EC. Studies have found a close relationship between As and coal combustion [58]. Previous research by Dai et al. [59] reported that coal burned in domestic stoves during winter is a major source of environmental sulfates, with sulfate reacting with ambient ammonia to form ammonium salts. The fourth factor, traffic emissions, is primarily composed of OC and EC, along with minor amounts of NH_4_^+^, Cl^−^, Mg^2+^, K, Ca, and Pb. Cl and K have been reported to come from vehicular emissions [60]. The online data lack components of Al and Si, and Ca can be considered to come from road dust [61]. Studies have indicated that motor vehicles may be a significant source of NH_4_^+^ in urban areas [62]. The fifth factor, characterized by high concentrations of NO_3_^−^, SO_4_^2−^, and NH_4_^+^, is identified as secondary inorganic aerosol (SIA), consistent with the typical mixed SIA factor reported in other studies [63,64]. The sixth factor comprises components such as Na^+^, Mg^2+^, Ti, Ni, EC, OC, Cl^−^, and SO_4_^2−^. Na^+^, Mg^2+^, Cl^−^, and SO_4_^2−^ are typical components of sea salt [65], and the Bohai Gulf is located approximately 30 km southeast of the site. Sea salt has previously been considered a source of particulate matter for Tianjin and the surrounding coastal and inland areas. Ti, Ni, EC, and OC are likely derived from the mixing and aging with other particles along the transport pathways. In general, SIA was responsible for the largest fraction of PM_2.5_ mass on average (32.7%), followed by traffic emission (26.2%), coal combustion (24.1%), industry emissions (7.5%), dust (6.7%), and sea salt (1.9%). On average, traffic emission appears to be the predominant primary source of PM_2.5_ for the measurement campaign.

### 3.3. Health Risk of PM_2.5_ and VOCs

**Figure 3:**
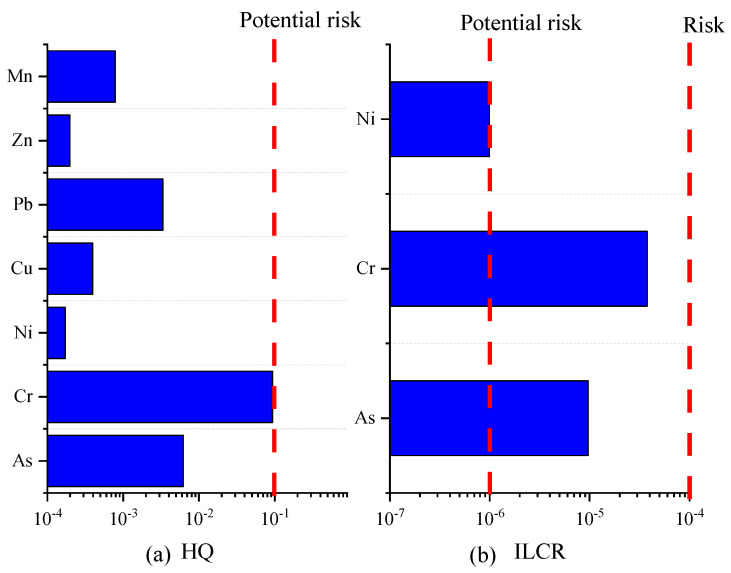
Health risks of trace metals in Tianjin. The result of hazard quotient (**a**) and incremental lifetime cancer risk (**b**).

Figure 3 presents an assessment of the health risks posed by particulate matter and VOCs in Tianjin City for the year 2019. The non-carcinogenic risk, quantified by the hazard quotient (HQ), associated with trace metals in particulate matter—namely As, Cr, Ni, Cu, Pb, Zn, and Mn—is detailed in Figure 3a. The HQ values for these metals are ordered as follows: Cr > Mn > As > Cu > Pb > Zn > Ni, ranging from 1.7 × 10^−4^ to 9.4 × 10^−2^. With all individual HQ values significantly below unity and the cumulative HQ also below the threshold of 1, it is evident that the non-carcinogenic risks are well within safe limits, indicating no cumulative non-carcinogenic effects from exposure to these trace metals. The carcinogenic risk, expressed as the incremental lifetime cancer risk (ILCR), for the selected trace metals (As, Cr, and Ni) in particulate matter is depicted in Figure 3b. The ILCR values for Cr, As, and Ni are 3.8 × 10^−5^, 3.8 × 10^−6^, and 9.9 × 10^−7^, respectively, with chromium and arsenic presenting a higher risk in the order of Cr > As > Ni. The ILCR benchmarks indicate that a value below 1 × 10^−6^ is considered to carry no carcinogenic risk, a value between 1 × 10^−4^ and 1 × 10^−6^ suggests a potential carcinogenic risk, and a value exceeding 1 × 10^−4^ indicates a definite carcinogenic risk. The ILCR for chromium and arsenic falls within the range of potential risk, while nickel’s ILCR is below the threshold for negligible risk. This signifies a latent carcinogenic risk to the residents of Tianjin from exposure to chromium and arsenic. The aggregate ILCR, positioned between 1 × 10^−4^ and 1 × 10^−6^, underscores a certain degree of health hazard and a potential carcinogenic threat due to long-term exposure via the respiratory pathway.

**Figure 4:**
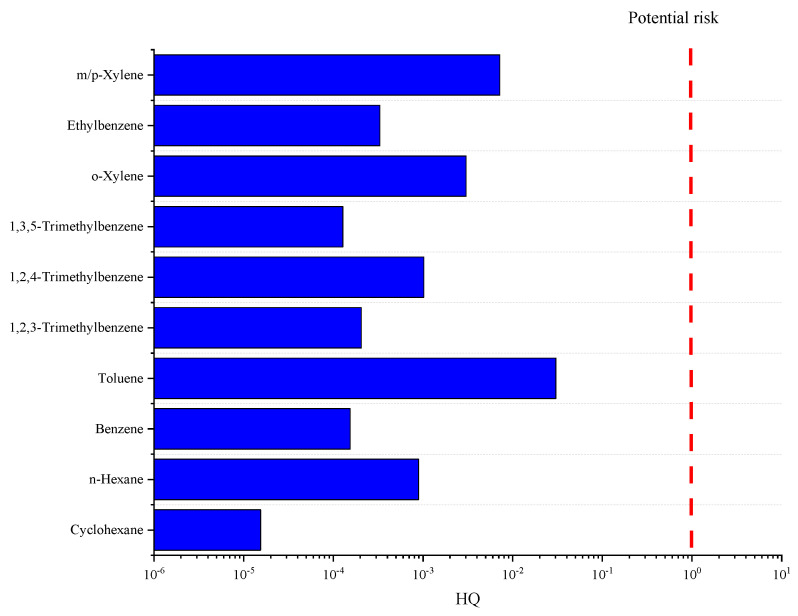
Hazard quotient of VOCS in Tianjin.

In the realm of VOCs, a health risk assessment was conducted on harmful species such as n-hexane, cyclohexane, and aromatic hydrocarbon compounds, with the non-carcinogenic risk (HQ) portrayed in Figure 4. The HQ values for these species fluctuate between 1.5 × 10^−5^ and 3.6 × 10^−2^, culminating in a total Hazard Index (HI) of 0.08. An HI value below the safety threshold of 1 demarcates the absence of a cumulative non-carcinogenic impact. Furthermore, the carcinogenic risk of benzene, a prominent VOC, was calculated. The carcinogenic threshold of VOCs is the same as that of heavy metals. Benzene’s carcinogenic risk, at 6 × 10^−6^, lies within the bracket of potential risk, signifying a latent carcinogenic threat to human health due to long-term environmental exposure.

### 3.4. Health Risk of Source

**Figure 5:**
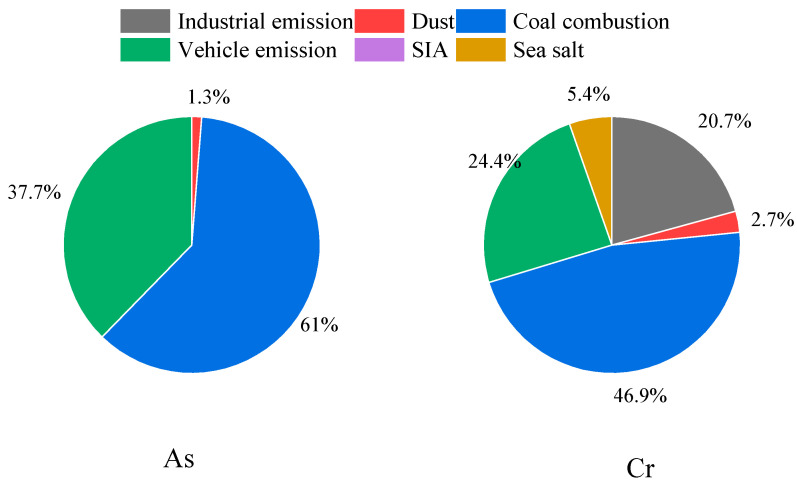
Sources of trace metals with health risk (As and Cr).

**Figure 6:**
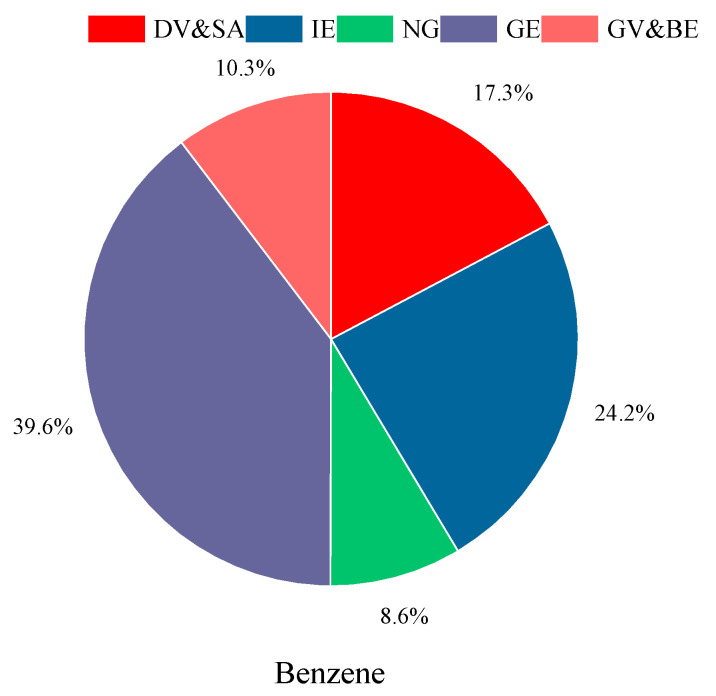
Sources of VOCs with health risk (benzene).

Drawing upon the outcomes of source apportionment analyses for PM_2.5_ and VOCs, this study has conducted a comprehensive assessment of the non-carcinogenic and carcinogenic health risk potentials associated with As and Cr, as well as benzene. The delineation of these risk sources is depicted in the accompanying Figure 5 and Figure 6.

The non-carcinogenic health risk potential for arsenic is predominantly linked to emissions from coal combustion, which accounts for a substantial 61% of the total. Vehicle emissions also contribute significantly, with a 37.7% share. Notably, contributions from dust sources are minimal, at approximately 1.3%, while the impacts of industrial emissions, secondary formation, and marine aerosols are found to be negligible. In the case of chromium, the non-carcinogenic health risk potential is primarily driven by coal combustion, which leads with a 46.9% contribution. Vehicle emissions follow closely, constituting 24.4% of the total risk. Industrial emissions are a significant factor, contributing 20.7%. The influence of marine aerosols and dust sources is comparatively minor, at 5.4% and 2.7%, respectively. It is evident that the control of emissions from coal combustion and vehicular sources is pivotal in mitigating the non-carcinogenic health risk potentials of heavy metals such as As and Cr in particulate matter.

Turning our attention to benzene, a known carcinogen, its health risk potential is attributed to several key sources (Figure 6). The most significant contributor is the volatilization of fuels, which encompasses 48.2% of the total risk, with GE accounting for 39.6% and natural gas (NG) for 8.6%. Emissions from vehicles and the use of solvents are also substantial, contributing 27%, with DV&SA collectively accounting for 17.3%, and GV&BE contributing 10%. Additionally, IE still contributes a notable 24.2%. The findings underscore the critical need for targeted strategies to control the volatilization of fuels, curtail industrial emissions, and regulate vehicular and solvent emissions. Such measures are essential in reducing the carcinogenic health risk potential of benzene in VOCs, thereby safeguarding public health and contributing to the broader goals of air quality management.

## 4. Conclusions

We conducted an analysis of the atmospheric pollution characteristics in Tianjin City using a 2019 online dataset of PM_2.5_ and VOCs. The atmospheric particulate matter in Tianjin is predominantly composed of water-soluble ions, complemented by carbonaceous components, with nitrates emerging as the most significant constituent. Within the VOC profile, alkanes are the most abundant, succeeded by alkenes and aromatic hydrocarbons, with compounds such as propane, ethane, ethylene, toluene, and benzene exhibiting notably high concentrations.

Utilizing receptor modeling techniques, we resolved the pollution sources of particulate matter and VOCs into distinct categories. For particulate matter, six major source types were identified—industrial emission, vehicle emissions, coal combustion, dust, secondary sources, and sea salt—with secondary sources, vehicular emissions, and coal combustion identified as the principal contributors. For VOCs, seven major source types were delineated—industrial emission, combustion emission, NG volatilization, gasoline evaporation, gasoline vehicles and biogenic emission, diesel vehicle and solvent application, and others—with industrial processes, NG volatilization, gasoline evaporation, and gasoline vehicles and biogenic emission recognized as the primary source categories.

In conclusion, we assessed the health impacts of the particulate matter and VOC components within the region. The non-carcinogenic risks associated with particulate matter and VOCs were determined to be negligible. Nevertheless, certain trace metals, namely As and Cr, along with benzene among the VOCs, were found to pose a potential carcinogenic risk, indicating the need for targeted monitoring and mitigation strategies to safeguard public health. This study conducted a risk attribution analysis for components in PM_2.5_ and VOCs that pose health risks. The findings reveal that the primary sources of the hazardous constituents As and Cr in PM_2.5_ are coal combustion and vehicular emissions, which account for a striking 99% and 70% of the total contributions to As and Cr, respectively. For the VOCs, the source of the high-risk component benzene is predominantly attributed to fuel evaporation, industrial emissions, and vehicular exhaust. These sources are identified as the principal contributors to the health risks associated with benzene exposure. The implications of these findings are clear: by intensifying the regulation and control of toxic and harmful components in coal combustion, industrial processes, and vehicular emissions, there is a significant potential to reduce the health risks posed by PM_2.5_ and VOCs. Such measures are essential for safeguarding public health and achieving broader air quality management objectives.

## Data Availability

The data are provided in tables and figures directly within the manuscript, and raw data are available via e-mail upon request to the corresponding author.

## Supplementary Materials

The following supporting information can be downloaded at https://www.mdpi.com/article/10.3390/toxics12090622/s1, Figure S1: Factor profile of VOCs; Figure S2: Factor profile of PM_2.5_; Table S1: The values of RfC and IUR of the VOCs; Table S2: The values of RfD and CSF of the trace metals; Table S3. The concentrations and percentages of the VOC species.
