# Supplementary material for: Characteristic Analysis and Health Risk Assessment of PM2.5 and VOCs in Tianjin Based on High-Resolution Online Data
Source: Toxics. 2024 Aug 23;12(9):622. doi: 10.3390/toxics12090622 (PMC11436045; doi:10.3390/toxics12090622)
Supplement: Supplementary file 1 [file toxics-12-00622-s001.zip › Supplementary materials.pdf]

Characteristics analysis and health risk assessment of $PM_{2.5}$ and VOCs in Tianjin based on high resolution online data

Yanqi Huangfu[1,2], Feng Wang[1,2,3], Qili Dai[1,2], Danni Liang[1,2], Guoliang Shi[1,2], Yinchang Fen[1,2,*]

1.State Environmental Protection Key Laboratory of Urban Ambient Air Particulate Matter Pollution Prevention and Control, College of Environmental Science and Engineering, Nankai University, Tianjin, 300350, China

2.China Meteorological Administration-Nankai University (CMA-NKU) Cooperative Laboratory for Atmospheric Environment-Health Research, College of Environmental Science and Engineering, Nankai University, Tianjin, 300350, China

3 School of Environmental Science and Safety Engineering, Tianjin University of Technology, Tianjin 300384, China.

* Correspondence: fengyc@nankai.edu.cn (Yinchang Feng)

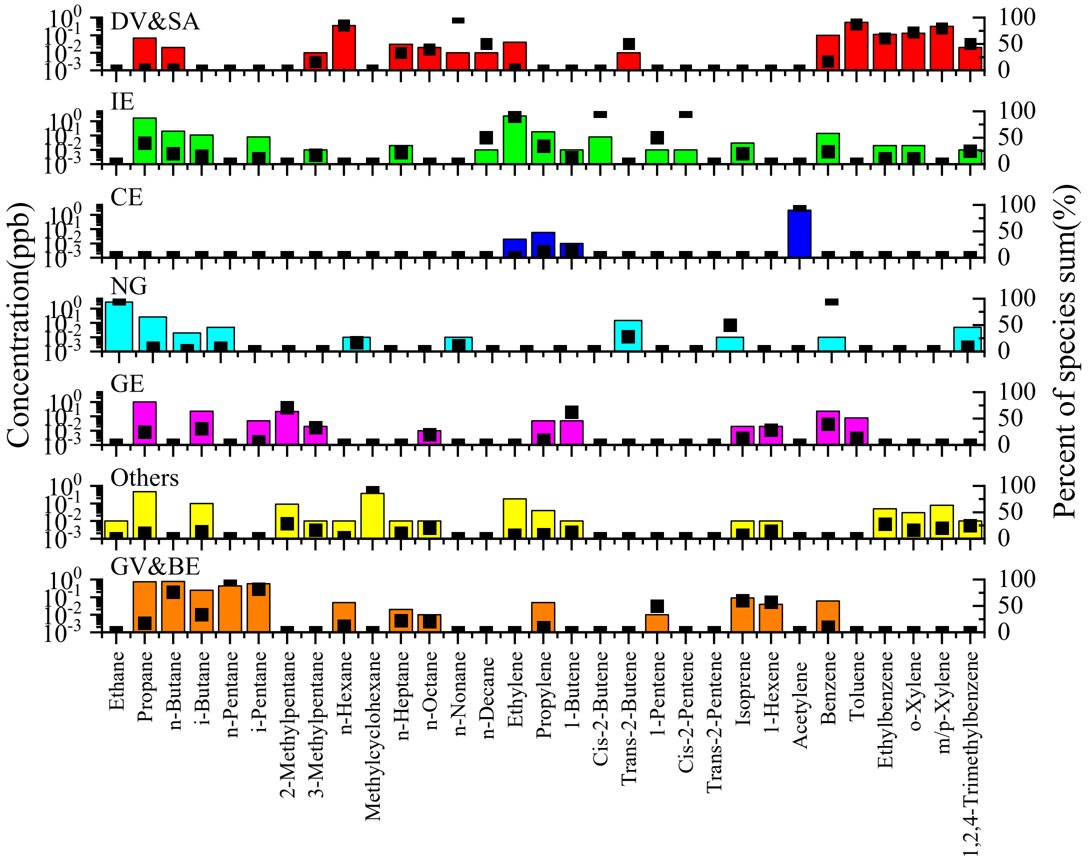

Figure.S1 Factor profile of VOCs.

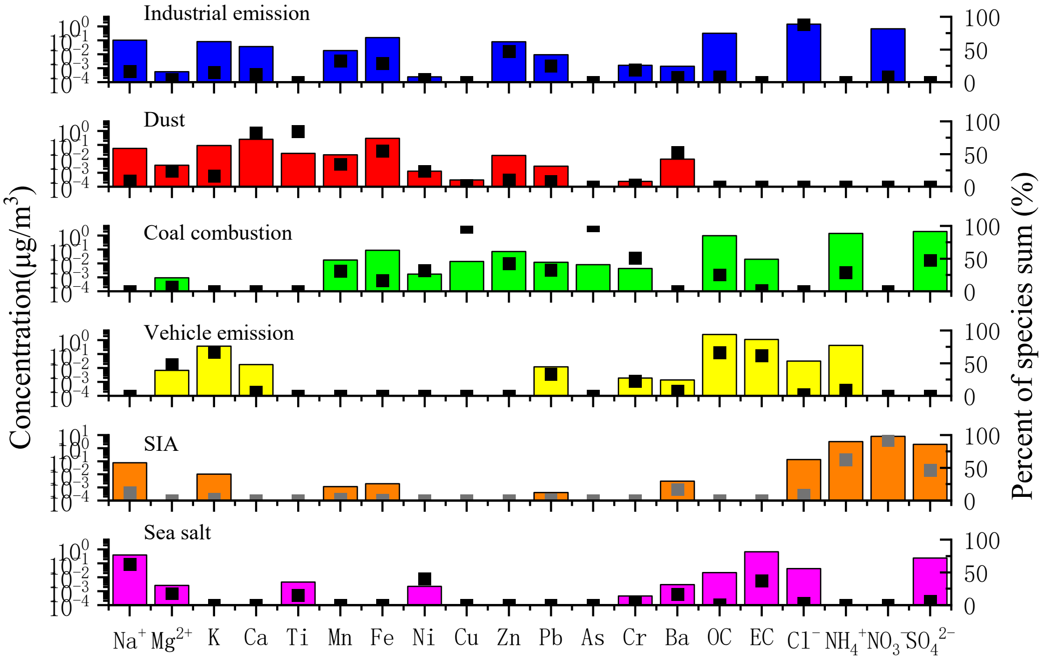

Figure.S2 Factor profile of $PM_{2.5}$

Table S1 The value of RfC, IUR of VOCs.

Species	RfC (mg/m^3)	IUR ($m^3/\mu g$)
Cyclohexane	6	-
n-Hexane	7.0×10^{-1}	-
Benzene	5	7.80×10^{-6}
Toluene	3.0×10^{-2}	-
1,2,3-Trimethylbenzene	6.0×10^{-2}	-
1,2,4-Trimethylbenzene	6.0×10^{-2}	-
1,3,5-Trimethylbenzene	6.0×10^{-2}	-
o-Xylene	0.1	-
Ethylbenzene	1	-
m/p-Xylene	1.0×10^{-1}	-

* Inhalation unit risk (IUR) (μg/m3) and reference concentrations (RfCs, mg/m^3). All the parameters in TableS1 were obtained from the Integrated Risk Information System (IRIS), United States Agency for Toxic Substances and Disease Registry, or California Office of Environmental Health Hazard Assessment (OEHHA)

Table S2 The value of RfD, CSF of trace metals.

Species	RfD (mg/kg·d)	CSF $(mg/kg\cdot d)^{-1}$
As	3.0×10^{-4}	15.1
Cr	2.8×10^{-5}	42
Ni	2.0×10^{-2}	8.4×10^{-1}
Cu	4.0×10^{-2}	-
Pb	3.5×10^{-3}	-
Zn	3.0×10^{-1}	-
Mn	2.4×10^{-2}	-

* RfD (mg/kg·day) is daily reference dose; CSF $(kg\cdot day/mg)^{-1}$ is the cancer slope factor. All the parameters in TableS2 were obtained from U.S. EPA.

Table S3 The concentrations and percentages of VOC species.

Species	AC (ppbv)	SD (ppbv)	Per (%)	Min (ppbv)	Max (ppbv)
Alkanes	14.9	14.9	61.8	0.5	176.5
Ethane	2.9	4.0	11.8	0.0	71.9
Propane	4.6	4.6	18.9	0.2	53.2
n-Butane	1.2	1.7	4.8	0.0	19.7
i-Butane	0.9	0.9	3.6	0.0	9.2
2,2-Dimethylbutae	0.1	0.2	0.3	0.0	5.8
2,3-Dimethylbutane	0.1	0.4	0.4	0.0	7.1
n-Pentane	0.5	0.8	2.0	0.0	7.7
i-Pentane	0.8	1.1	3.4	0.0	13.6
Cyclopentane	1.2	4.0	4.8	0.0	78.4
2-Methylpentane	0.4	0.7	1.8	0.0	21.9

3-Methylpentane	0.1	0.1	0.3	0.0	3.1
Methylcyclopentane	0.8	1.8	3.2	0.0	20.2
2,4-Dimethylpentane	0.1	0.4	0.4	0.0	5.7
2,3-Dimethylpentane	0.0	0.1	0.1	0.0	0.9
2,3,4-Trimethylpentane	0.1	0.1	0.4	0.0	1.6
2,2,4-Trimethylpentane	0.1	0.2	0.6	0.0	3.2
n-Hexane	0.5	0.7	2.0	0.0	7.4
3-Methylhexane	0.1	0.1	0.3	0.0	1.8
2-Methylhexane	0.0	0.1	0.2	0.0	2.0
Cyclohexane	0.1	0.1	0.3	0.0	2.5
Methylcyclohexane	0.4	1.4	1.7	0.0	19.3
n-Heptane	0.1	0.1	0.4	0.0	2.0
2-Methylheptane	0.0	0.2	0.1	0.0	10.6
3-Methylheptane	0.0	0.3	0.2	0.0	9.5
n-Octane	0.1	0.1	0.2	0.0	4.9
n-Nonane	0.0	0.0	0.1	0.0	1.0
n-Decane	0.0	0.2	0.2	0.0	3.5
Alkenes	4.8	5.0	19.8	0.1	39.9
Propene	2.6	3.5	10.9	0.0	27.2
trans-2-Butene	0.8	1.2	3.1	0.0	14.6
1-Butene	0.1	0.1	0.4	0.0	2.5
cis-2-Butene	0.2	0.6	0.9	0.0	6.6
trans-2-Pentene	0.1	0.3	0.2	0.0	10.4
1-Pentene	0.0	0.1	0.1	0.0	1.1
cis-2-Pentene	0.0	0.1	0.1	0.0	4.1
Isoprene	0.0	0.1	0.1	0.0	1.4
1-Hexene	0.5	1.4	2.1	0.0	22.3
Ethylene	0.5	1.4	1.9	0.0	22.1
Aromatic hydrocarbons	2.3	2.6	9.5	0.0	22.0
Benzene	0.7	0.6	2.7	0.0	4.3
Toluene	0.7	0.9	2.8	0.0	11.7
Ethylbenzene	0.2	0.3	0.9	0.0	3.2
m,p-Xylene	0.2	0.3	0.8	0.0	3.1
Styrene	0.5	0.7	1.9	0.0	8.3
o-Xylene	0.0	0.0	0.0	0.0	0.6
i-Propylbenzene	0.0	0.0	0.0	0.0	0.2
n-Propylbenzene	0.0	0.1	0.0	0.0	5.1
m-Ethyltoluene	0.0	0.1	0.1	0.0	1.1
p-Ethyltoluene	0.0	0.0	0.0	0.0	2.1
1,3,5-Trimethylbenzene	0.0	0.0	0.0	0.0	0.5
o-Ethyltoluene	0.0	0.1	0.1	0.0	1.1
1,2,4-Trimethylbenzene	0.0	0.0	0.0	0.0	0.6
1,2,3-Trimethylbenzene	0.0	0.0	0.0	0.0	0.5
m-Diethylbenzene	0.0	0.0	0.0	0.0	0.5

p-Diethylbenzen	2.3	2.6	9.5	0.0	22.0
Acetylene	2.2	3.5	9.0	0.0	79.2
TVOC	24.1	22.6	-	0.9	209.4

* AC is the average con concentrations of VOCs, SD represents the standard deviation, Per refers to the percentage of species in TVOCs. Min and Max represent the minimum and maximum values of VOCs. All the data in TableS3 were obtained from Nankai University Air Quality Research Supersite (NKAQRS).
